# Supplementary material for: Chromothripsis during telomere crisis is independent of NHEJ, and consistent with a replicative origin
Source: Genome Res. 2019 May;29(5):737–49. doi: 10.1101/gr.240705.118 (PMC6499312; doi:10.1101/gr.240705.118)
Supplement: Supplemental Material [file supp_gr.240705.118_Supplemental_file_1.zip › contigs/annotated_contigs/DB89/contig.2.DB89_length_361_mean_cov_5.81717451524.docx]

**DB89_length_361_mean_cov_5.81717451524**

```
GGAGGCTGAGGCAGGAGAATGCCGTGAATCCAGGAGGCGGAGCTTGCAGTGAGCTGAGATCACACCACTGTACTCCAGCCTGGGCGACA
 >chr7:65651386-65651503 - E=6e-44 p=0e+00
GAGCAAG|ATTCCGTCTCAAAAAAAAAAA|ACCCAAACCCCATTCTCATACCCGCTCCACAGCCTCTGCTCTGGCCTCTGCAATATAAT
        >chr19:36376898-36377166 - E=7e-143
CACACAAAGAGCCCCCATCCCAGAAAGCCCTTGGACCCATCACAGCCCCCATAGCTAGGAACCTTTCATGGACCACAGACCGAATGTAC

ATGTTAGCACATTCCGGGGCTCCAGCCAATGTCTCCACCCCAAAACTTAAGGCTTAAACCAACACCCTTTTAATTATTTCTTTTCTTTT

TTCTTTT
```
